# Plant Growth Promotion, Phytohormone Production and Genomics of the Rhizosphere-Associated Microalga, *Micractinium rhizosphaerae* sp. nov.

**DOI:** 10.3390/plants12030651

**Published:** 2023-02-01

**Authors:** Francisco Quintas-Nunes, Pedro R. Brandão, Maria T. Barreto Crespo, Bernard R. Glick, Francisco X. Nascimento

**Affiliations:** 1 iBET, Instituto de Biologia Experimental e Tecnológica, Apartado 12, 2781-901 Oeiras, Portugal; 2 Instituto de Tecnologia Química e Biológica António Xavier, Universidade Nova de Lisboa, Av. da República, 2780-157 Oeiras, Portugal; 3 Department of Biology, University of Waterloo, Waterloo, ON N2L 3G1, Canada

**Keywords:** microalgae, *Micractinium*, plant growth, phytohormones, rhizosphere, genomics

## Abstract

Microalgae are important members of the soil and plant microbiomes, playing key roles in the maintenance of soil and plant health as well as in the promotion of plant growth. However, not much is understood regarding the potential of different microalgae strains in augmenting plant growth, or the mechanisms involved in such activities. In this work, the functional and genomic characterization of strain NFX-FRZ, a eukaryotic microalga belonging to the *Micractinium* genus that was isolated from the rhizosphere of a plant growing in a natural environment in Portugal, is presented and analyzed. The results obtained demonstrate that strain NFX-FRZ (i) belongs to a novel species, termed *Micractinium rhizosphaerae* sp. nov.; (ii) can effectively bind to tomato plant tissues and promote its growth; (iii) can synthesize a wide range of plant growth-promoting compounds, including phytohormones such as indole-3-acetic acid, salicylic acid, jasmonic acid and abscisic acid; and (iv) contains multiple genes involved in phytohormone biosynthesis and signaling. This study provides new insights regarding the relevance of eukaryotic microalgae as plant growth-promoting agents and helps to build a foundation for future studies regarding the origin and evolution of phytohormone biosynthesis and signaling, as well as other plant colonization and plant growth-promoting mechanisms in soil/plant-associated *Micractinium.*

## 1. Introduction

Microalgae are a ubiquitous and diverse group of prokaryotic and eukaryotic photosynthetic organisms inhabiting various aquatic and terrestrial environments worldwide. These microorganisms have been traditionally used as food/feed sources and have recently gained considerable interest, as some of them are able to synthesize and accumulate several compounds of industrial and commercial interest, such as lipids and fatty acids, sugars, proteins, and carotenoids among others [1,2,3,4,5]. As a consequence of these characteristics, the microalgae industrial production sector has grown immensely in the last few decades and is set to expand in the near future [2,6].

Despite being traditionally linked to aquatic environments, microalgae also play key roles in soil and in association with plants. Microalgae (both cyanobacteria and eukaryotic species) are common inhabitants of natural and agricultural soils and play indispensable roles in soil C and N fixation, nutrient cycling, detoxification, maintenance of soil structure and overall soil health [7,8,9,10]. Additionally, recent work has demonstrated that microalgae are important members of the plant microbiome and may greatly impact plant growth, development, and stress resistance [11]. Several reports have demonstrated that not only cyanobacteria, but also eukaryotic microalgae such as members of the Chlorellaceae family are able to promote the growth of several crop plants (e.g., tomato, wheat, soybean, pepper) [12,13,14,15], and this is accomplished using microalgae cell cultures or extracts and diverse plant inoculation strategies (e.g., foliar or soil application) [11]. *Chlorella* cell-free supernatants can also reduce the onset of shoot and flower senescence with potential benefits for increasing flower shelf-life [16]. In addition, there is evidence regarding the plant protection abilities of several microalgae strains, based on their ability to directly inhibit the growth of pathogens [17,18] as well as to induce plant defense responses that lead to increased plant immunity [19]. For example, Lee and colleagues have demonstrated that the foliar application of *Chlorella fusca* induced defense responses in *Arabidopsis thaliana* which led to increased protection against *Pseudomonas syringae* pv. tomato DC3000 infection. This response was induced by the D-lactic acid secreted into the supernatant of *C. fusca,* which acted as a defense priming agent [19]. Altogether, these results are bringing new insights and considerable interest to the use of microalgae as biofertilizers, biostimulants and biocontrol agents for several agricultural applications.

Several studies indicate that the plant growth-promoting effects of microalgae such as *Chlorellaceae* are linked with its ability to synthesize a wide range of growth-inducing compounds such as organic acids, vitamins and phytohormones, including auxins (e.g., indole-3-acetic acid -IAA), cytokinins, and others [11]. Nevertheless, not much is understood regarding the potential of different microalgae strains in potentiating plant growth, nor the mechanisms involved in such activities. To address this challenge, we have isolated several microalgae strains from the rhizosphere of several plants, including the microalgal strain, NFX-FRZ, belonging to the *Micractinium* genus, which was isolated from the roots of a wild *Ficus* spp. plant obtained from a Portuguese soil. Despite the presence of soil moisture, the occurrence of observable green spots in the plant root tissues revealed a strong interaction between the microalgae and the plant host, suggesting a more intricate interaction between the two eukaryotes. In this work, we present a detailed functional and genomic characterization of strain NFX-FRZ, including its ability to synthesize a wide range of compounds and phytohormones, and demonstrate its effects in plant growth promotion.

## 2. Results and Discussion

### 2.1. Characterization of Micractinium rhizosphaerae sp. nov.

**Figure 1:**
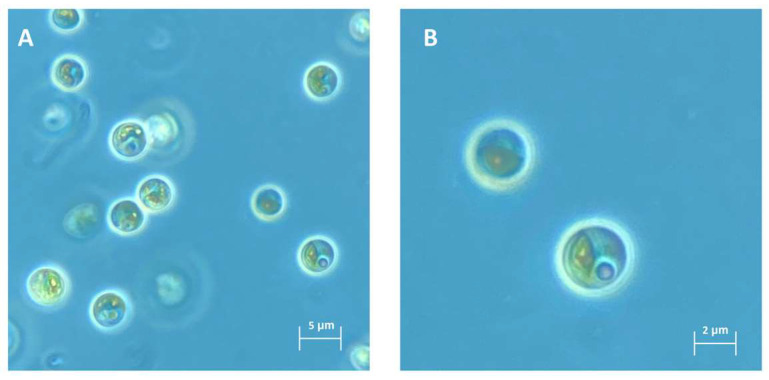
Morphological characteristics of strain NFX-FRZ cultivated in algae culture broth.

The microalga strain NFX-FRZ presented a spherical form, with cells of approximately 3.5 × 3.2 μm (Figure 1A,B). The non-motile cells did not present any coverage or appendages and reproduced asexually by autospores. A single cup-shaped chloroplast containing a well-defined pyrenoid was found (Figure 1A,B). The cells contained well defined starch and lipid bodies (Figure 1A,B).

**Figure 2:**
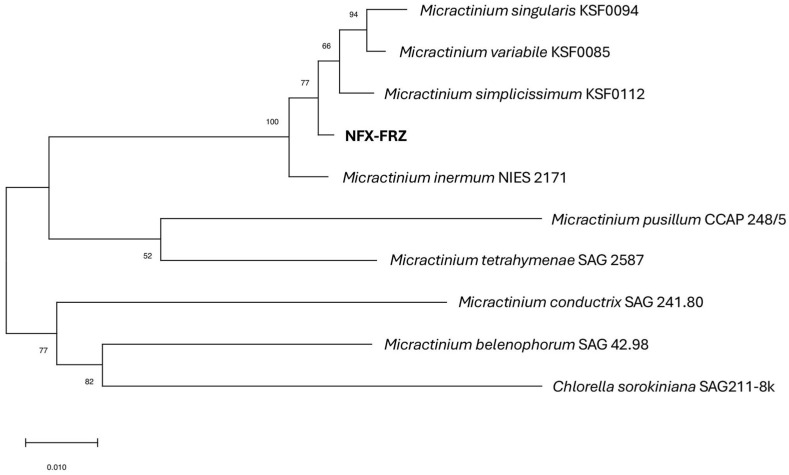
Phylogram based on *Micractinium* 18S-ITS1-5.8S-ITS2 regions (2477 bp). The evolutionary history was inferred by using the maximum likelihood method and Tamura-Nei model. The tree with the highest log likelihood (−5991.31) is shown. The percentage of trees in which the associated taxa clustered together is shown next to the branches. A discrete gamma distribution was used to model evolutionary rate differences among sites (5 categories (+G, parameter = 0.2992)). The rate variation model allowed for some sites to be evolutionarily invariable ([+I], 62.93% sites). The tree is drawn to scale, with branch lengths measured in the number of substitutions per site.

BLAST analysis showed that the strain NFX-FRZ 18S-ITS1-5.8S-ITS2 region presented increased identity to the 18S-ITS1-5.8S-ITS2 region of *Micractinium* species. Phylogenetic analysis based on the 18S-ITS1-5.8S-ITS2 region confirmed that strain NFX-FRZ is a member of the *Micractinium* genus, grouping close to the *M. inermum* species (Figure 2). However, strain NFX-FRZ formed an independent cluster in the phylogram, indicating that it does not belong to the *M. inermum* species. These data suggest that strain NFX-FRZ belongs to a novel species, tentatively termed *Micractinium rhizosphaerae* sp. nov.

**Figure 3:**
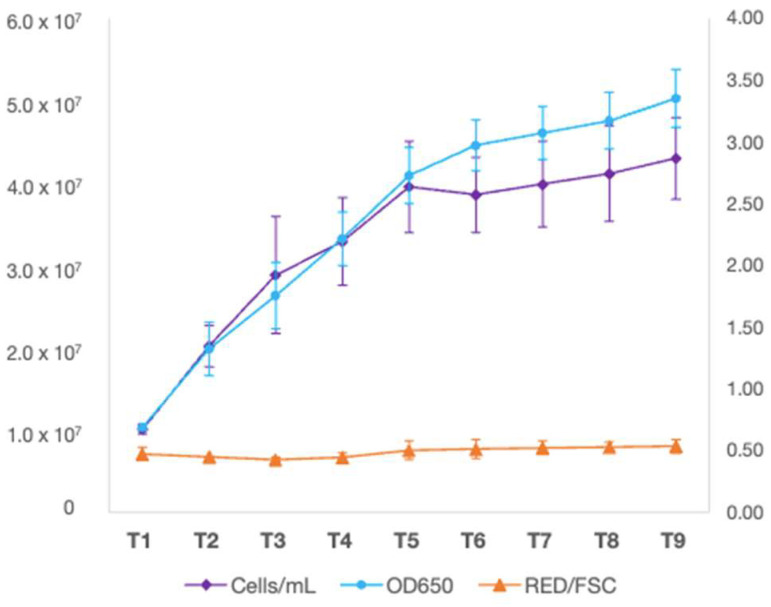
*Micractinium rhizosphaerae* NFX-FRZ growth dynamics when cultivated in algae culture broth (1.5×) for 9 days.

*M. rhizosphaerae* NFX-FRZ autotrophic growth on algae culture broth led to a maximum of 4.3 × 10^7^ cells /mL at 9 days after inoculation (DAI) (Figure 3). The ratio of RED/FSC parameters (relative fluorescence/relative cell size) remained stable throughout the experiment, indicating a stable photosynthetic activity and variation in cell size (Figure 3).

### 2.2. Micractinium rhizosphaerae NFX-FRZ Promotes Tomato Plant Growth and Actively Binds to Plant Roots

**Figure 4:**
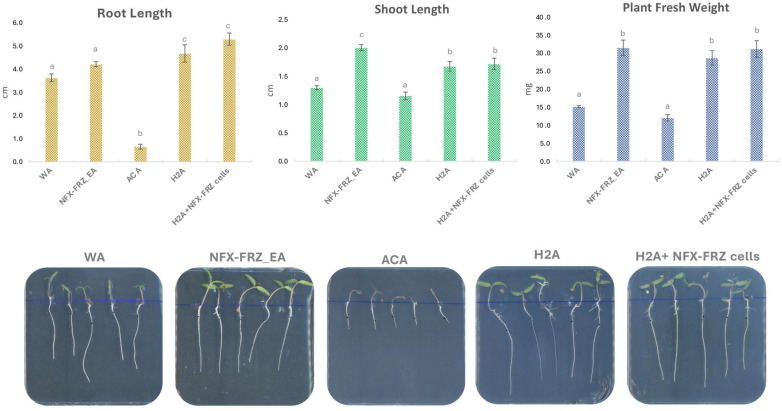
Results obtained from the tomato plant growth promotion assays. Different letters above the bars represent statistical differences (*p* < 0.05). WA—water agar; NFX-FRZ_EA—NFX-FRZ exudates agar; ACA—algae culture agar (1.5X algae culture broth + 0.5% Agar); H2A—Hoagland’s No. 2 basal salt agar.

Plate assays revealed the beneficial and plant growth promotion effects of *M. rhizosphaerae* NFX-FRZ, especially its exudates (Figure 4). Tomato plants grown in NFX-FRZ exudate agar plates displayed increased development compared to tomato plants grown in water agar or algae culture agar (Figure 4). The NFX-FRZ exudate agar plants presented a significantly increased shoot elongation and total fresh weight compared to plants in water agar (Figure 4). Moreover, the roots, shoots and overall fresh weight were significantly increased in plants cultivated in NFX-FRZ exudate agar compared to plants grown in algae culture agar (Figure 4). Similar growth dynamics were observed between plants grown in NFX-FRZ Exudate Agar and plants grown in Hoagland’s No. 2 basal salt agar (control), notwithstanding the fact that the shoots of plants cultivated in NFX-FRZ exudate agar were significantly more elongated and developed (Figure 4), indicating differentiated growth properties of the NFX-FRZ exudates. A detailed metabolomic analysis of NFX-FRZ exudates is presented and discussed below.

Curiously, the growth of tomato plants grown in algae culture agar was negatively affected so that their overall growth was inhibited compared to the plants grown in water agar and Hoagland’s No. 2 basal salt agar controls (Figure 4). It is possible that the nitrate and phosphate sources and concentrations (NaNO_3_; K_2_HPO_4_; 1.500 g/L and 0.500 g/L, respectively) present in the algae culture agar led to the inhibition of plant growth (especially sodium nitrate). These nutrient sources and concentrations are different from those found in the Hoagland’s No. 2 basal salt mixture (Ca(NO_3_)_2_; KNO_3_; (NH_4_)_3_PO_4_; 0.656 g/L, 0.606 g/L and 0.115 g/L, respectively). Importantly, plant growth-promoting NFX-FRZ exudates from microalgae cultivations in algae culture broth presented the same nutrient sources and concentrations as found in algae culture agar. The microalgae growth in the algae culture broth possibly led to a decrease in the inhibitory nutrient concentrations that affected tomato plant growth. Ultimately, microalgae such as *M. rhizosphaerae* NFX-FRZ can be useful to treat other nutrient-rich waters presenting inhibitory effects on plant growth (e.g., industrial waste waters), leading to a better use of water resources for agricultural applications. In this regard, the use of other *Micractinium* strains for the treatment of wastewaters and removal of nitrate and phosphate is well documented [20,21].

**Figure 5:**
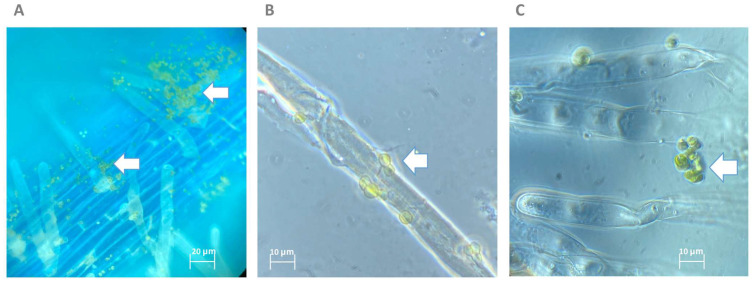
Microscopy observations of the tomato root colonization abilities of *Micractinium rhizosphaerae* NFX-FRZ. Arrows indicate the presence of the NFX-FRZ strain. (**A**) overview of the primary root tissue; (**B**) NFX-FRZ cells attached to plant root hairs; (**C**) clusters of NFX-FRZ cells bound to plant root hairs.

The tomato plants grown in Hoagland’s No. 2 basal salt agar and inoculated with NFX-FRZ cells showed a slight increase in root elongation and fresh weight compared to plants grown in Hoagland’s No. 2 basal salt agar and receiving PBS (Figure 4), but the differences were not statistically significant. The results suggest that plant inoculation with NFX-FRZ cells may also lead to plant growth promotion; however, the short incubation period (4 days) may not be sufficient to observe more profound effects. In fact, microscopic observations showed that *M. rhizosphaerae* NFX-FRZ effectively attached to tomato roots (Figure 5A) and root hairs (Figure 5B) in the root maturation and the initial elongation areas (~2 cm from the root shoot junction, initial inoculation point), but was not detected in developing elongation and cell division areas (~2–7 cm). Since *M. rhizosphaerae* NFX-FRZ does not have motility, its root colonization properties and, consequently, its direct plant growth-promoting effects may take more time to be established. Curiously, *Micractinium* and other members of the *Chlorellaceae* family are known inhabitants and mutualistic partners of several highly motile protozoans [22,23,24]. It is possible that these microalgae–protozoa interactions play a key role in the transport of microalgae cells throughout soil and rhizosphere environments, thereby promoting microalgae colonization. Nonetheless, the results obtained herein directly show the ability of *M. rhizosphaerae* NFX-FRZ to independently bind to external plant root tissues. Moreover, the microscopy observations showed that when attached to plant tissues, *M. rhizosphaerae* NFX-FRZ often formed “clusters” of cells bound to one another (Figure 5C), a situation which was not detected when the microalgae strain was grown singly and in liquid growth media, suggesting specific adaptations of *M. rhizosphaerae* to the rhizosphere environment.

### 2.3. Metabolomic Analysis of Micractinium rhizosphaerae NFX-FRZ Exudates Revealed the Presence of Several Phytohormones and Plant Growth-Promoting Compounds

#### 2.3.1. General Untargeted Metabolomic Analysis

An untargeted metabolomic analysis was conducted to characterize the plant growth-promoting exudates of *Micractinium rhizosphaerae* NFX-FRZ. The analysis led to the detection of 5563 resolved *m*/*z* peaks in the negative ionization mode (Table S1), from which 115 were identified (Table S2), and 3497 resolved *m*/*z* peaks in the positive ionization mode (Table S3), from which 146 were identified (Table S4). The top 15 produced compounds with increased relative peak areas detected and identified in each of the negative and positive ionization modes are presented in Table 1. Organic acids such as tartaric acid, lactic acid, azelaic acid, malic acid, pyruvic acid, stearic acid were amongst the top 15 compounds with increased relative peak areas detected in the negative ionization mode (Table 1). In the positive ionization mode, the top 15 compounds with increased relative peak areas are thiazole-containing compounds such as benzothiazole and 2-acetylthiazole, which were widely prevalent alongside the carbohydrate levoglucosan; organic acids, such as 1-methoxy-1H-indole-3-acetonitrile, indoleacetic acid (IAA), hydrocinnamic acid; amino acids, such as phenylalanine; and nucleosides such as uridine (Table 1). The results obtained demonstrate that *M. rhizosphaerae* NFX-FRZ exudates are of chemical complexity, indicating a wide range of metabolic activities involved in the biosynthesis of several organic compounds of biological significance. For example, organic acids such as tartaric and malic acid play a role in plant protection against stress [25,26]. Lactic acid produced by Chlorella acts as a plant defense priming agent [19]. Benzothiazole derivatives are known to present plant growth regulation activities, similar to those induced by cytokinins and auxins [27].

#### 2.3.2. Phytohormones

Several phytohormones were detected in the *M. rhizosphaerae* NFX-FRZ exudates (Table 2). Amongst these, the most predominant was the auxin, indole-3-acetic acid (IAA) (Table 2), which is widely known for its vital impact in plant growth and development [28]. The results suggest that the plant growth-promoting effects of the NFX-FRZ exudates could be induced by the microalgae synthesized IAA. Several microalgae, including Chlorella (a close relative of *Micractinium*), also synthesize and secrete IAA to the external growth media [29,30,31,32]. Despite its role as a plant growth regulator, IAA is also an inducer of strong physiological responses in several microalgae, impacting cell size, membrane permeability, photosynthetic potential and the accumulation of lipids and other secondary compounds [30,33,34], suggesting an ancient role for IAA in the regulation, growth, and development of Chlorophyta. The *M. rhizosphaerae* NFX-FRZ exudates were collected during its maximal growth phase, indicating a role for IAA in this organism’s own development, possibly through the regulation of several of the processes mentioned above.

The phytohormone salicylic acid (SA) (2-hydroxybenzoic acid), as well as benzoic acid (BA) and 2,4-dihydroxybenzoic acid (2-4-HBA) were found in the NFX-FRZ exudates (Table 2). SA is a phenolic compound that regulates several aspects of plant growth, development, and stress responses [35]. In microalgae such as Chlorella, SA and BA act as signaling molecules that regulate cell growth, increasing carbon assimilation and activating DNA-replicating enzymes [36]. Moreover, SA helps to decrease the negative effects of abiotic stress in microalgae growth and improve the accumulation of lipids and carotenoids [37,38,39].

The *M. rhizosphaerae* NFX-FRZ exudates also contained linoleic, linolenic acid and several forms of jasmonic acids (JA) (epi-jasmonic acid, methyl jasmonic acid, methyl dihydrojasmonic acid) (Table 2 and Table S2). Jasmonic acids are a class of compounds that are derived from the oxidative metabolism of alpha-linolenic acid and play significant roles in plant defense responses and plant growth regulation [40]. The production and secretion of jasmonates by several microalgae has been previously described [31,32]. Moreover, the application of exogenous JAs has been found to lead to increased lipid accumulation in Chlorella [39].

Although in lower concentrations, abscisic acid (ABA) was detected in the *M. rhizosphaerae* NFX-FRZ exudates (Table 2), which is consistent with previous reports indicating the widespread production and secretion of ABA by several microalgae species, including Chlorella [31,32,41]. In plants, ABA plays essential roles in multiple physiological processes as well as in responses to abiotic and biotic stresses [42]. In microalgae, the production of ABA is also related to the presence of stress conditions and is thought to also be involved in the regulation of microalgae growth and [41,43]. The exogenous application of ABA also leads to the accumulation of lipids in *Chlorella* [39,44].

### 2.4. Micractinium rhizosphaerae NFX-FRZ Genomic Properties

The genome of *M. rhizosphaerae* NFX-FRZ had a predicted size of 68.28 Mbp and a 65.3% GC content. The nuclear genome was represented by 1497 contigs and the chloroplast and mitochondrial genomes were each represented by a single circular element, of 120.1 Kbp, 34.2% GC and 74.4 Kbp, 30.3% GC, respectively (Table 3). The deduced genomic properties of *M. rhizosphaerae* NFX-FRZ were somewhat similar to the reported genomic properties of *Micractinium conductrix* SAG 241.80 (Table 3), the unique representative genome described of a member of the *Micractinium* genus [22].

The *M. rhizosphaerae* NFX-FRZ genome annotation indicated 14,335 CDS present in the NFX-FRZ nuclear genome. BUSCO analysis revealed that the obtained nuclear proteome contained 99.5% of the Chlorophyta core proteins (genes) (98.6% complete genes, [single-copy 97.5%, duplicated 1.1%], 0.9% fragmented, and 0.5% missing). These results agree with the BUSCO values obtained in the analysis of the published proteome [22] of *M. conductrix* which has SAG 241.80 (96.0% complete genes, [single-copy 88.4%, duplicated 7.6%], 0.7% fragmented, and 3.3% missing), indicating the increased quality of the *M. rhizosphaerae* NFX-FRZ genome annotation.

The functional annotation of the *M. rhizosphaerae* NFX-FRZ nuclear genome was performed using GHOSTKOALA; 5085 entries were annotated (Table S5). Most of the annotated CDS were related to genetic information processing, carbohydrate metabolism and signaling and cellular processes, followed by other metabolic functions (Table 3 Importantly, the data obtained indicate similar functional annotations in both *M. rhizosphaerae* NFX-FRZ and *M. conductrix* SAG 241.80 nuclear genomes, leading to similar metabolic modules (KEGG) found both strains (Table S6). However, some significant differences exist between these strains, and some modules were only detected in *M. rhizosphaerae* NFX-FRZ (Table S6). This is the case for the ceramide, sphingosine, and N-glycan precursor biosynthesis modules, of which all genetic components were found in strain NFX-FRZ, but not in *M. conductrix* SAG 241.80 (Table S6). Since most of these modules are involved in the biosynthesis of cell wall components, these results suggest different cell wall properties in *M. rhizosphaerae* NFX-FRZ compared to *M. conductrix* SAG 241.80.

### 2.5. Genomic Insights into Micractinium rhizosphaerae NFX-FRZ Phytohormone Production Abilities

#### 2.5.1. Auxins, IAA

Several biosynthetic pathways for IAA production have been identified in microorganisms such as bacteria [45] as well as in higher plants [46]. These pathways are mostly dependent on the amino acid tryptophan, which is the main precursor of IAA. Despite being mediated by different genetic elements in different organisms, the IAA biosynthesis pathways mostly occur through four intermediates: indole-3-pyruvate (IPyA), indole-3-acetonitrile (IAN), indole-3-acetamide (IAM) and tryptamine (TRA) [45].

The NFX-FRZ genome contains all the genetic elements involved in the de novo biosynthesis of tryptophan (Tables S5–S7). Moreover, several aminotransferases, an aromatic aminotransferase (ISS/VAS1) homolog (g82.t1), a pyruvate decarboxylase (PDC1) (g10007.t1), a YUCCA-like enzyme (g6702.t1), and an indole-3-acetaldehyde oxidase (AAO2) encoding gene (g7700.t1) were detected in the genome of strain NFX-FRZ and represent the main genetic elements involved in IAA biosynthesis via the IPyA pathway. Several amino acid monooxygenases and four amidase-encoding genes, including two homologs of *Arabidopsis* AMI1 genes (g3851.t1; g6165.t1) involved in the conversion of IAM to IAA, were also found. The genes encoding an aromatic-L-amino-acid/L-tryptophan decarboxylase (g10395.t1; g10403.t1), converting tryptophan to tryptamine, and a monoamine oxidase (g13669.t1), converting tryptamine to indole-3-acetaldehyde, were also detected in the genome (Tables S5 and S7). Altogether, the obtained results suggest that *M. rhizosphaerae* NFX-FRZ synthesizes IAA via the IPyA, IAM and TRA pathways. It is not unusual for a plant growth-promoting organisms to contain several pathways for the synthesis of IAA. In fact, several bacterial strains possess multiple IAA biosynthetic pathways [47]. The advantage of multiple IAA biosynthetic pathways in a single strain is that if the organism loses elements of any one pathway, that organism is still able to synthesize IAA [48].

In addition to the genetic elements involved in IAA biosynthesis, several genes involved in IAA transport and signaling were also identified in the NFX-FRZ genome (Tables S5 and S7). The genome harbors a PILS (PIN-like auxin transporter) homolog (g13509.t1) involved in the regulation of intracellular auxin concentrations [49]; a homolog of the Medicago truncatula LAX4 gene (g1607.t1) that encodes an auxin carrier protein involved in proton-driven auxin influx [50]; and two ABP1 gene homologs (g10599.t1, g10691.t1) encoding auxin-binding proteins that act as receptors for endogenous auxins [51]. Similar results have been described for Chlorella sorokiniana UTEX 1230, which contains multiple genetic elements involved in IAA biosynthesis and auxin transport and signaling [52].

#### 2.5.2. Salicylic Acid

Two pathways involved in SA biosynthesis in plants have been described: the phenylalanine-based pathway which leads to benzoic acid (BA) and ultimately SA formation, and the chorismate pathway in which chorismate is transformed into isochorismate that is then converted to SA [53]. Genomic analysis revealed that the NFX-FRZ genome contains the CS, CM and ADT genes encoding chorismate synthase, chorismate mutase and arogenate dehydratase involved in phenylalanine biosynthesis via chorismate (Tables S5 and S7). However, a PAL gene encoding phenylalanine ammonia lyase was not detected in the NFX-FRZ genome, suggesting that SA biosynthesis via the phenylalanine pathway is not active in this strain. In addition, single isochorismate synthase (ICS) genes were not found in the NFX-FRZ genome; however, a PHYLLO homolog was detected (g1643.t1). The PHYLLO protein is a multifunctional enzyme required for phylloquinone (vitamin K1) biosynthesis and is composed of several fused genes, including the menF homolog which encodes isochorismate synthase [54]. The obtained results indicate that *M. Rhizosphaerae* NFX-FRZ does not possess a dedicated isochorismate biosynthetic pathway like the one that evolved in higher plants [54], further suggesting that its isochorismate and SA biosynthesis activities are linked and possibly dependent on the biosynthesis of phylloquinone, which is vital for photosystem I function [55]. Despite the knowledge regarding the function of ICS and its role in the biosynthesis of SA, not much is known regarding the enzymatic conversion of isochorismate to SA in plants. In bacteria, the conversion of isochorismate to SA is mediated by an isochorismate pyruvate lyase (IPL) enzyme; however, no homologs of this enzyme have been detected in plants [53]. The genome *M. rhizosphaerae* NFX-FRZ does not harbor IPL homologs; however, two isochorismatase family proteins (g13049.t1, g13681.t1) were detected.

Homologs of the NPR (non-expressor of pathogenesis-related) genes involved in plant SA signaling were not detected in the NFX-FRZ genome, suggesting the possibility of alternative SA signaling mechanisms in *Micractinium*.

#### 2.5.3. Jasmonic Acid

In plants, jasmonic acid (JA) biosynthesis is dependent on the precursor alpha-linolenic acid, occurs in two major steps occurring within the chloroplast and the peroxisome, and involves the action of several enzymes linked to lipid/fatty acid metabolism including lipases (PLA, DAD), lipoxygenases (LOX), allene oxide synthase (AOS), allene oxide cyclase (AOC), 12-oxo-phytodienoic acid (OPDA) reductase (OPR), 3-oxo-2-(2′-[Z]-pentenyl)cyclopentane-1-hexanoic acid→(OPC)-8:0 CoA ligase (OPCL1), acyl-CoA-oxidase (ACX), enoyl-CoA hydratase/3-hydroxyacyl-CoA dehydrogenase (MFP2) and 3-ketoacyl-CoA thiolase homologs (KAT1, PED1) [56].

*M. rhizosphaerae* NFX-FRZ, as well as most Chlorellaceae microalgae, contains the genetic machinery for the biosynthesis of polyunsaturated fatty acids such as alpha-linolenic acid (Tables S5–S7). Additionally, genomic analysis showed the presence of multiple lipase-encoding genes (Tables S5 and S7), a LOX2 homolog gene (g134.t1), three OPR3 like-genes (g470.t1, g631.t1, g13537.t1), a fatty acid CoA-ligase gene (g7562.t1), five ACX genes (g155.t1, g10375.t1, g11084.t1, g12245.t1, g12811.t1) two KAT1 genes (g1114.t1, g1115.t1) and one PED1 gene homolog (g2526.t1) (Tables S5 and S7). The data therefore suggest that *M. rhizosphaerae* NFX-FRZ can synthesize JAs via OPDA, despite the absence of plant-like AOS and AOS homologs. A previous study has demonstrated that allene epoxydes are naturally unstable and could be directly converted non-enzymatically to OPDA [57]. Alternatively, other cytochromes p450, epoxide hydrolase, and cyclase encoding genes present in the NFX-FRZ genome (Tables S5 and S7) could be responsible for OPDA biosynthesis. In addition, the JAR1 gene involved in JA amino acid conjugation was not found in the NFX-FRZ genome.

Despite of the presence of JA biosynthesis genes in *M. rhizosphaerae* NFX-FRZ, no homologs of the plant genes involved in JA signaling (e.g., COI1, JAZ, MYC) were detected, further suggesting that in *Micractinium,* the JA signaling, and JA-induced responses are somewhat differently regulated, using other genetic machinery which remains to be discovered. These results are consistent with the data presented by Han [58] demonstrating that the evolution of JA signaling mechanisms occurred in land plants.

#### 2.5.4. Abscisic Acid

Plant ABA biosynthesis occurs via carotenoid oxidation and is mediated by several enzymes involved in the carotenoid biosynthesis process [59] such as zeaxanthin epoxidase (ZEP) and 9-cis-epoxycarotenoid dioxygenase (NCED), enzymes which are involved in the synthesizes of important ABA precursors (e.g., xanthoxin). Later steps involve the action of ABA2, which encodes a short chain dehydrogenase/reductase-like enzyme and abscisic aldehyde oxidase (AAO) that lead to the final formation of ABA [59]. The genome of *M. rhizosphaerae* NFX-FRZ contains all the genes encoding the enzymes necessary for carotenoid biosynthesis via the methylerythritol phosphate (MEP) pathway, including genes involved in beta-carotene, zeaxanthin and violaxanthin biosynthesis (Tables S5–S7). Additionally, two ZEP genes (g2574.t1, g3683.t1), a NCED homolog (g3879.t1), ABA2 homologs (g1194.t1, g1552.t1, g3099.t1) and a AAO homolog (g7700.t1) were found in the NFX-FRZ genome (Tables S5 and S7), suggesting that strain NFX-FRZ synthesizes ABA via xanthoxin.

Genomic analysis showed that homologs of the genes involved in the plant ABA signaling cascade such as PYR-like receptors, were not found in the NFX-FRZ genome. This data suggests that ABA signaling mechanisms in *Micractinium* evolved differently from plants.

## 3. Material and Methods

### 3.1. Strain Isolation, Identification, and Characterization

The NFX-FRZ strain was isolated from the roots of a wild *Ficus* tree in Varelinha, Ferreira do Zêzere, Portugal. A portion of the plant roots was washed with sterile water, and 50 µL of washing solution was plated in algae culture agar (algae culture broth: ammonium chloride, 0.05 g/L; calcium chloride, 0.058 g/L; dipotassium phosphate, 0.25 g/L; ferric chloride, 0.003 g/L; magnesium sulfate, 0.513 g/L; sodium nitrate, 1 g/L; and agar, 15 g/L). The plate was incubated in the presence of light (90 μmol/s/m^2^ white LED light) and an average temperature of 22 °C. Individual axenic microalgae colonies were isolated and subsequently maintained in algae culture agar. The strain NFX-FRZ was identified based on its genomic data, including the 18S-ITS1-5.8S-ITS2 region (described below) and characterized by its morphological characteristics, which were observed using a Zeiss AX10 microscope.

### 3.2. Phylogenetic Analysis

The available 18S-ITS1-5.8S-ITS2 regions of *Micractinium* species were obtained from the NCBI database (https://www.ncbi.nlm.nih.gov/ (accessed on 01 November 2022)) and were aligned using MUSCLE [60]. Phylogenetic analyses were conducted using MEGA X [61]. The maximum likelihood method and the best model determined based on the lowest Bayesian information criterion score were used. A total of 500 replicates (bootstrap) were performed for each analysis.

### 3.3. Growth Kinetics under Autotrophic Conditions

Axenic autotrophic growth kinetic experiments were conducted under laboratory conditions using 2 L Schott flasks receiving 1.2 L of 1.5X algae culture broth (pH 7). The autotrophic cultivations were performed at 22 °C, with an aeration rate of 0.2 L/min (compressed air filtered through a Millex-FG 50 mm 0.2 µm PTFE filter and dispersed through a DURAN^®^ Gas distribution tube of 33cD, d = 6 mm and porosity = 1), in the presence of light (90 μmol/s/m^2^ white LED light) with a day/night cycle of 16:8 h. The cultivation experiments received an initial inoculum solution of approximately 1 × 10^6^ cells/mL (~100 mL of inoculum in 1.2 L total cultivation volume). A total of six replicates (flasks) were conducted.

### 3.4. Tomato Plant Growth Promotion Assays

Plant growth promotion assays were conducted in square agar plates (12 × 12 cm) using tomato as the host plant and using different treatment approaches. A total of five treatments were conducted, corresponding to (1) plants grown in water agar (WA) (0.75% agar, pH 6.9) plates; (2) plants grown in NFX-FRZ exudate agar (0.75% agar, pH 6.8); (3) plants grown in algae culture agar (ACA) (ACB 1.5x, 0.75% agar, pH 7.0); (4) plants grown in Hoagland 2 agar (H2A) (, 0.75% agar, pH 6.9) and inoculated with sterile phosphate-buffered saline (PBS); and (5) plants grown in H2A and inoculated with a NFX-FRZ solution (2 × 10^7^ cells/mL in PBS).

To understand the plant growth-promoting effects of strain NFX-FRZ exudates, agar plates were created using exudates collected from microalgae autotrophic growth experiments (described above) at maximal growth conditions (T5, ~4 × 10^7^ microalgae cells/mL). The microalgae exudates were collected by centrifugation, directly filtered using a sterile 0.2 µm PTFE filter and directly mixed with a sterile agar solution to a final concentration of 0.75% agar. The inoculation and root colonization effects were evaluated following the application of 10 μL of a microalgae solution (2 × 10^7^ cells/mL in PBS).

Tomato seeds were surface sterilized by immersion in a 70% ethanol solution for 1 min, 10 min in a sodium hypochlorite solution (1%), followed by five washing steps with sterile distilled water. The disinfected seeds were placed in 1% water agar plates and incubated for three days in the dark at 24 °C. The germinated seedlings were then used for the plate growth promotion assays. A total of three square plates containing five plants each were conducted for each treatment (total of 15 plant replicates per treatment). The square plates were sealed using parafilm, the root portion covered with aluminum foil, and then incubated vertically to receive light directly in the developing shoot (90 μmol/s/m^2^ white LED light) in a day/night cycle of 16:8 h. The plates were incubated at an average temperature of 21 °C for four days. After this period, several plant growth parameters were evaluated, including root elongation, shoot elongation, and plant fresh weight. Elongation measurements were conducted using ImageJ software. Statistical analyses were performed by ANOVA and post hoc Tukey’s test using PAST v.4.11 software [62].

### 3.5. Untargeted Metabolomic Analysis of NFX-FRZ Exudates

Microalgae supernatants were obtained from growth kinetic experiments under autotrophic conditions. The exudates were obtained at T5 (5 days after inoculation) after the microalgae exponential growth phase. Briefly, 20 mL of microalgae solution were aseptically removed from the flasks and immediately centrifuged at 7000 rpm for 10 min at 4 °C in an Eppendorf 5430R centrifuge. The supernatant obtained was then filtered using an axenic 0.2 µm PTFE filter and immediately frozen. The supernatants from three biological replicates were sent for untargeted metabolomic analysis by Creative Proteomics (New York, NY, USA) using in-house developed protocols. Briefly, 1 mL of sample was lyophilized to dryness and then dissolved in 500 μL of 80% methanol. All samples were vortexed for 60 s, followed by sonication for 30 min at 4 °C. Then, each sample was kept at −20 °C for 1 h, and after that, period samples were centrifuged at 12,000 rpm and 4 °C for 15 min. Finally, 200 μL of supernatant and 5 μL of DL-o-chlorophenylalanine (1 mg/mL) were transferred to vials for LC-MS analysis. The separation was performed by ACQUITY UPLC (Waters) combined with Q Exactive MS (Thermofisher) and screened with ESI-MS (+ and − ionization modes). The LC system was comprised of ACQUITY UPLC HSS T3 (100 × 2.1 mm × 1.8 μm) with ACQUITY UPLC (Waters). The mobile phase was composed of solvent A (0.05% formic acid water) and solvent B (acetonitrile) with a gradient elution (0–1 min, 5% B; 1–12 min, 5–95% B; 12–13.5 min, 95% B; 13.5–13.6 min, 95–5% B; 13.6–16 min, 5% B). The flow rate of the mobile phase was 0.3 mL min*^−^*^1^. The column temperature was maintained at 40 °C, and the sample manager temperature was set at 4 °C.

The obtained chromatograms were analyzed, and metabolite identification performed with the Compound Discoverer software affiliated with Thermo Q Exactive. The normalized values for each metabolite were obtained by dividing the peak area of each metabolite by the sum of all metabolite areas, and then multiplying by one million.

### 3.6. Genome Sequencing and Analysis

Strain NFX-FRZ total genomic DNA was extracted using a Norgen Biotech Plant/Fungi DNA extraction kit following the manufacturer’s instructions. The DNA obtained was analyzed and sent to Macrogen Inc., (Seoul, Republic of Korea) for library construction (Illumina TruSeq Nano DNA library) and sequencing (Illumina HiSeqX, 150 × 2 bp, paired-end). The reads obtained were further trimmed using Trimmomatic [63] default parameters (sliding window trimming, average quality = 25). A total of 97,902,861 reads were used in the final assembly, which was performed using the SPADES DNA-Seq De Novo Assembler [64]. The assembly was further polished using Pilon [65] and QUAST [66], and repeat sequences were identified with RepeatMasker 4.09 (https://www.repeatmasker.org/ (accessed on 1 November 2022). Organellar genomes were assembled using GetOrganelle [67] using standard parameters. Complete plastid and mitochondrial genomes were obtained. The nuclear ribosomal RNA (18S-ITS1-5.8S-ITS2) region was identified in the genome contigs based on BLAST analysis [68] using the Geneious software [69]. The NFX-FRZ nuclear genome, complete chloroplast and mitochondrial genomes were submitted to the DDBJ/ENA/GenBank under the accession number JAPTJF000000000.

The nuclear genome annotation of NFX-FRZ was conducted using WebAugustus [70]. Briefly, a gene annotation training set was created based on the *M. conductrix* SAG 241.80 genome (NCBI assembly, ASM224581v2) and transcripts (RNAseq data from PRJNA290385) which was further used to annotate the NFX-FRZ genome. Additionally, the obtained chloroplast and mitochondrial genomes of strain NFX-FRZ were annotated using GeSeq [71] based on the complete sequences of *Chlorella* (*Micractinium*) sp. ArM0029B (KF554427.1, KF554428.1) [72], the closest relative in terms of sequence similarity.

The completeness of genomes and the respective genome annotations were analyzed in the gVolante website [73] using BUSCO v.5 [74] and the Chlorophyta ortholog set as reference.

Genome functional annotations were performed using GHOSTKOALA [75] and BLASTp analysis (standard parameters) against the UNIPROT database [76] which were conducted in the Geneious software.

## 4. Conclusions

Strain NFX-FRZ, isolated from the roots of a plant in Portugal, presents unique genotypic characteristics, including signatures that allow its classification as a novel species termed *Micractinium rhizosphaerae* sp. nov. Metabolomic analysis revealed that the NFX-FRZ strain had the ability to grow autotrophically in inorganic media, synthesizing and exuding a wide range of plant growth-promoting compounds, including phytohormones such as IAA, SA, JA, and ABA. Moreover, *M. rhizosphaerae* NFX-FRZ effectively colonized plant root tissues and actively promoted plant growth, thus acting as a plant-growth-promoting algae (PGPA). These characteristics may be explored in future applications aimed at increasing plant growth and plant stress resistance.

Genomic analysis showed that *M. rhizosphaerae* NFX-FRZ had a unique genomic structure that contained multiple genes involved in the biosynthesis of phytohormones. Most of the phytohormone biosynthesis genes detected were homologs to those of plants, indicating an ancient origin for phytohormone biosynthesis genes in Chlorophyta. Nevertheless, most of the plant genes involved in phytohormone signaling were absent from the *M. rhizosphaerae* NFX-FRZ genome, thus suggesting that this organism utilized different phytohormone signaling mechanisms. Future studies are necessary to understand the genetic mechanism involved in phytohormone biosynthesis and signaling in *Micractinium* and other members of the *Chlorellaceae* family.

This work provides new insights regarding the relevance of eukaryotic microalgae as plant-growth-promoting agents and lays a foundation for future studies regarding the origin and evolution of phytohormone biosynthesis and signaling, as well as other plant colonization and plant growth-promoting mechanisms in *Micractinium*.

## Figures and Tables

**Table 1:** Top 30 compounds identified in NFX-FRZ exudates (15 from each of negative and positive ionization modes).

Name	Molecular Weight	*m*/*z*	RT [min]	Average Normalized Peak Area	Ionization Mode
Tartaric acid	150.02	149.01	0.89	10,113.57	-
Stearic acid	284.27	283.26	11.43	1366.32	-
Phthalic acid	166.03	165.02	3.98	1069.89	-
Gingerol	294.18	293.18	8.37	964.23	-
3-methyl-2-oxovaleric acid	130.06	129.05	3.66	897.09	-
Lactic acid	90.03	89.02	1.23	797.01	-
Azelaic acid	188.10	187.10	5.36	677.12	-
Malic acid	134.02	133.01	0.88	659.34	-
Pyruvic acid	88.01	87.01	0.90	638.25	-
2-Isopropylmalic acid	176.07	175.06	3.68	611.98	-
3-Hydroxy-3-methylglutaric acid	162.05	161.04	1.59	559.06	-
10-oxo-8-decenoic acid	184.11	183.10	5.94	434.27	-
2,4-Dihydroxybenzoic acid	154.03	153.02	0.88	405.99	-
Indole	117.06	116.05	3.27	389.51	-
Salicylic acid	138.03	137.02	5.67	351.88	-
Benzothiazole	135.01	136.02	6.37	32,851.00	+
1-Methoxy-1H-indole-3-acetonitrile	186.08	94.05	0.72	26,810.47	+
2-Acetylthiazole	135.01	136.02	6.37	13,111.33	+
Indole-3-acetic acid	186.08	94.05	0.72	8808.64	+
Levoglucosan	127.01	128.02	0.72	2797.73	+
S-Aminoethyl-cysteine	219.03	110.52	14.65	1331.66	+
Hydrocinnamic acid	162.05	163.06	1.26	1234.93	+
Sedanolide	164.06	83.04	0.73	994.99	+
Traumatin	150.07	301.14	0.81	822.69	+
Phenylalanine	194.13	195.14	6.68	811.84	+
Sphinganine	212.14	213.15	6.67	672.22	+
Thymoquinone	165.08	166.09	2.40	368.78	+
Sulfamic acid	301.30	302.30	8.12	328.02	+
Pyroglutamic acid	164.08	165.09	5.21	253.56	+
Uridine	96.98	139.02	14.64	244.76	+

**Table 2:** Phytohormones detected in *M. rhizosphaerae* NFX-FRZ exudates.

Name	Molecular Weight	*m*/*z*	RT [min]	Average Normalized Peak Area	Ionization Mode
Indole-3-acetic acid	219.03	110.52	14.65	8808.64	+
3-Indoleacrylic acid	187.06	188.07	3.28	65.43	+
Salicylic acid	138.03	137.02	5.67	351.88	-
p-Hydroxysalicylic acid	154.03	153.02	0.88	405.99	-
Methyl dihydrojasmonate	226.16	225.15	9.27	173.19	-
Epi-jasmonic acid	210.13	211.13	7.64	142.77	+
Dihydrojasmonic Acid	212.14	211.13	7.39	53.98	-
Methyl Jasmonate	224.14	225.15	5.52	19.33	+
Abscisic acid	264.14	265.14	8.66	29.13	+

**Table 3:** Genomic properties of *M. rhizosphaerae* NFX-FRZ and *M. conductrix* SAG 241.80.

	*M. rhizosphaerae* NFX-FRZ	*M. conductrix* SAG 241.80
Nuclear genome size (Mbp)	68.3	60.8
Nuclear genome GC%	65.3%	67.4%
Chloroplast genome size (bp)	120,168	129,445
Chloroplast genome GC%	32.4	34.8
Mitochondrial genome size (bp)	74,426	75,014
Mitochondrial genome GC%	30.3	29.4
Nuclear CDS	14,335	10,070
Annotated CDS	5085	4566
GIP	1096 + 932	980 + 893
CM	390	359
SCP	373	321
EIP	237	168
CP	224	216
EM	198	177
AAM	190	167
MCV	175	184
LM	154	141
GBM	116	75
NM	94	88
MTP	43	39
MAA	37	34

GIP—genetic information processing; CM—carbohydrate metabolism; SCP—signaling and cellular processes; EIP—environmental information processing; CP—cellular processes; EM-energy metabolism; AAM—amino acid metabolism; MCV—metabolism of cofactors and vitamins; LM—lipid metabolism; GBM—glycan biosynthesis and metabolism; NM—nucleotide metabolism; MTP—metabolism of terpenoids and polyketides; MAA—metabolism of other amino acids.

## Data Availability

Genomic data are available in the DDBJ/ENA/GenBank under the accession number JAPTJF000000000.

## Supplementary Materials

The following supporting information can be downloaded at: https://www.mdpi.com/article/10.3390/plants12030651/s1; Table S1: Compounds detected in the negative ionization mode; Table S2: Compounds identified in the negative ionization mode; Table S3: Compounds detected in the positive ionization mode; Table S4: Compounds identified in the positive ionization mode; Table S5: NFX-FRZ GHOSTKOALA Functional annotation; Table S6: *Micractinium* KEGG Modules; Table S7: Comparison to the UNIPROT database (BLASTp results); Data: protein fasta DOI 10.5281/zenodo.7527345.
